# Extracellular vesicles of the Gram-positive gut symbiont *Bifidobacterium longum* induce immune-modulatory, anti-inflammatory effects

**DOI:** 10.1038/s41522-023-00400-9

**Published:** 2023-06-03

**Authors:** Noa Mandelbaum, Lihan Zhang, Shaqed Carasso, Tamar Ziv, Sapir Lifshiz-Simon, Irina Davidovich, Ishai Luz, Elliot Berinstein, Tal Gefen, Tomer Cooks, Yeshayahu Talmon, Emily P. Balskus, Naama Geva-Zatorsky

**Affiliations:** 1 Department of Cell Biology and Cancer Science, Rappaport Faculty of Medicine and Research Institute, Rappaport Technion Integrated Cancer Center (RTICC), Technion-Israel Institute of Technology, Haifa, 31096 Israel; 2 Department of Chemistry and Chemical Biology, Harvard University, Cambridge, MA USA; 3 Smoler Proteomics Center, Lokey Interdisciplinary Center for Life Sciences & Engineering, Technion-Israel Institute of Technology, Haifa, 3200003 Israel; 4 Department of Chemical Engineering and the Russell Berrie Nanotechnology Institute (RBNI), Technion-Israel Institute of Technology, Haifa, 3200003 Israel; 5 The Shraga Segal Department of Microbiology, Immunology and Genetics, Faculty of Health Sciences, Ben-Gurion University, Beer-Sheva, 84105 Israel; 6 Howard Hughes Medical Institute, Harvard University, Cambridge, MA USA; 7 Humans and the Microbiome, CIFAR, Toronto, Canada

**Keywords:** Bacteriology, Applied microbiology

## Abstract

The gut microbiota is now well known to affect the host’s immune system. One way of bacterial communication with host cells is via the secretion of vesicles, small membrane structures containing various cargo. Research on vesicles secreted by Gram-positive gut bacteria, their mechanisms of interaction with the host and their immune-modulatory effects are still relatively scarce. Here we characterized the size, protein content, and immune-modulatory effects of extracellular vesicles (EVs) secreted by a newly sequenced Gram-positive human gut symbiont strain - *Bifidobacterium longum* AO44. We found that *B. longum* EVs exert anti-inflammatory effects, inducing IL-10 secretion from both splenocytes and dendritic cells (DC)-CD4+ T cells co-cultures. Furthermore, the EVs protein content showed enrichment in ABC transporters, quorum sensing proteins, and extracellular solute-binding proteins, which were previously shown to have a prominent function in the anti-inflammatory effect of other strains of *B. longum*. This study underlines the importance of bacterial vesicles in facilitating the gut bacterial immune-modulatory effects on the host and sheds light on bacterial vesicles as future therapeutics.

## Introduction

In the past two decades, many studies demonstrated the profound effects of the gut microbiota on human physiology^1–3^, with a range of beneficial functions predominantly related to the maturation and modulation of the immune system^3–14^. Immune-modulatory bacteria were long identified by us and others^15–18^, yet, only a few bacterial-derived immune-modulatory molecules have been characterized^19–21^. Bacterial vesicles have gained interest as an entity that can interact with both bacterial and host cells^22^. Vesicles are membrane structures secreted by both Gram-negative and Gram-positive bacteria. The size of the vesicles varies from 20–300 nm, and they carry various cargo, including proteins (both membranal and cytoplasmic), peptidoglycan, nucleic acid, and toxins, as well as lipopolysaccharide (LPS, in Gram-negative bacteria). Vesicles interact with bacteria and host cells by internalizing and releasing their cargo. These interactions make vesicles ideal for long-distance molecular delivery either to neighboring bacteria or host immune cells. As such, bacterial vesicles can be exploited for potential therapeutics^23^. Although vesicles produced by Gram-negative bacteria have been studied since the ‘60 s^24^, the production of extracellular vesicles (EVs) by Gram-positive bacteria was demonstrated 30 years later^25^. While discovered in the ‘90 s, interest in the vesiculogenesis and immune-modulatory effects of Gram-positive EVs increased in the past decade^26,27^, still, with most of the focus being on pathogenic bacteria such as *Staphylococcus aureus*^28^, *Mycobacterium tuberculosis*^29^, and *Bacillus anthracis*^30,31^. A few studies showed that only metabolically active Gram-positive bacteria secret EVs, different from Gram-negative bacteria^32,33^, however, recent studies suggest that EVs are also secreted in a process of “bubbling cell death”^34^. To date, there is a limited understanding of the factors involved in the genetic regulation of vesiculogenesis and the membranal state of the bacteria that enables EVs release^26,35^. Amongst the Gram-positive gut microbiota members, the *Bifidobacterium* genus gained interest as it is known to degrade human milk oligosaccharides and is highly prevalent in the gastrointestinal tract of breastfed infants^36^ as well as in adults’ intestines^37^. Furthermore, species of the *Bifidobacterium* genus were found to affect both the innate and adaptive immune system, mostly with anti-inflammatory effects^16,38^, with a few effector molecules characterized to date^39,40^. While several extracellular molecules, such as the *Bifidobacterium bifidum* pili^40^ and exopolysaccharides of *Bifidobacterium breve*^39^, were shown to induce anti-inflammatory effects, the mechanisms through which these molecules interact with the host are not fully understood. An important member of the *Bifidobacterium* genus is *Bifidobacterium longum*. This species is highly abundant in the human gut, even amongst the *Bifidobacterium* species^41^. *B. longum* was found to have an anti-inflammatory effect in vitro on cell lines^42^, in vivo in murine models^43^, and most importantly, in clinical trials of inflammatory bowel diseases (IBD)^44^. These anti-inflammatory effects were attributed mostly to its abilities to reduce oxidative stress, downregulate the secretion of inflammatory cytokines and increase the short-chain fatty acids (SCFA) content in the gut^45^. However, the molecular mechanisms underlying its interactions with the host and the therapeutic effects are yet to be discovered. Several studies have highlighted EVs secreted by *Bifidobacterium* species as anti-inflammatory with potential use as adjuvants in allergy treatment^46,47^. For example, vesicles of *B. bifidum* were found to interact with dendritic cells (DCs), followed by differentiation of regulatory T cells (T-regs)^47^. Intriguingly, a recent study highlighted the potential of *B. longum* vesicles in alleviating food allergy through apoptosis induction in mast cells^46^. Although studies on the immune-modulatory effects of vesicles from both Gram-negative and Gram-positive gut symbionts are starting to emerge^26^ only a few discovered the molecules and mechanisms underlying these effects. Moreover, EVs from several strains of the same species were shown to induce distinct immune-modulatory effects with different mechanisms, highlighting the great potential of vesicles derived from the thousands of bacterial strains found in the human gut^48^. Here we demonstrate the immune-modulatory effects of gut bacterial vesicles produced by a newly sequenced and annotated *Bifidobacterium longum* strain AO44. Our results open a new avenue for future studies on the potential therapeutic effects of bacterial vesicles.

## Results

### *B. longum* AO44 chloroform fractions exert higher Interleukin-10 (IL-10) secretion by splenocytes compared to other fractions

**Fig. 1:**
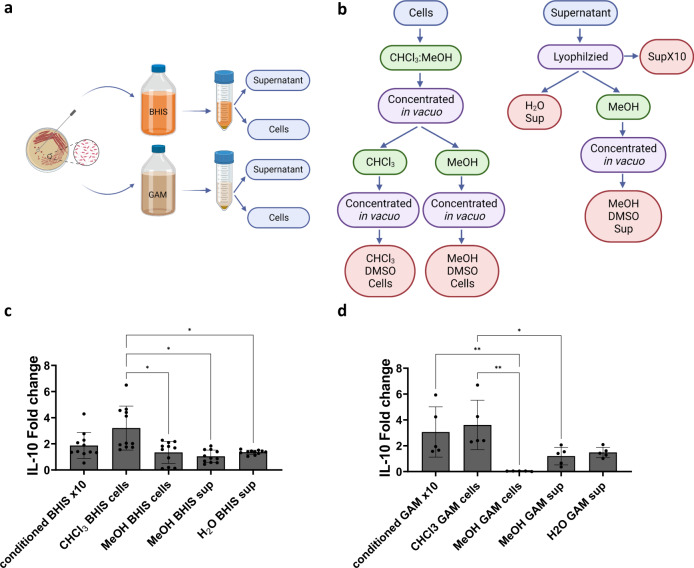
*B. longum* AO44 chloroform fractions induce splenocyte Interleukin-10 (IL-10) secretion. **a** Illustration of the bacterial growth and cells/supernatant separation. **b** Illustration of the bacterial conditioned media and cells chemical fractionation. **c** Fold change (compared to control) of IL-10 concentrations in splenocytes exposed to either of the chemical fractions generated from *B. longum* grown in BHIS media. **d** Fold change (compared to control) of IL-10 concentrations in splenocytes exposed to either of the chemical fractions generated from *B. longum* grown in GAM media. Each dot represents a technical repeat out of three independent experiments. Brown-Forsythe and Welch ANOVA, *P < 0.05 and **P < 0.01. Error bars represent s.d. CHCl_3_ fraction = hydrophobic organic fraction, MeOH fraction = hydrophilic organic fraction, H2O fraction = hydrophilic polar fraction, SupX10 = concentrated conditioned media. Figure 1a, b was created with BioRender.com.

DNA of *B. longum* strain AO44 was extracted, the whole genome was sequenced and deposited to GenBank, accession number PRJNA908295. To determine which bacterial entity stimulates an anti-inflammatory immune response (i.e., IL-10 secretion), chemical fractionation was performed using methanol/chloroform (MeOH/CHCl_3_) extractions. Both bacterial cells and conditioned media from *B. longum* AO44 grown in Gifu Anaerobic Medium (GAM) or Brain Heart Infusion supplemented (BHIS) were collected and hydrophobic/hydrophilic fractions were extracted (Fig. 1a, b). specific pathogen-free (SPF) mouse splenocytes were used to assess the immune-modulatory effects of each chemical fraction. Splenocytes were activated with anti-CD3 and supplemented with each bacterial fraction. SPF mouse splenocytes, activated with anti-CD3, enable studying the effects of bacterial entities on the whole population of immune cells found in the spleen. The CHCl_3_ fraction from bacteria grown in both GAM and BHIS exerted the highest IL-10 secretion by splenocytes compared to other fractions. Fold change from the control (splenocytes activated with anti-CD3) is presented (Fig. 1c, d). The immune-modulatory effects of the fraction of the concentrated (10x) conditioned media (i.e., the conditioned media) were similar to the CHCl_3_ fraction, indicating that a bacterial entity that exists in both fractions contains the active immune-modulatory component.

### *B. longum* AO44 EVs isolation and characterization

**Fig. 2:**
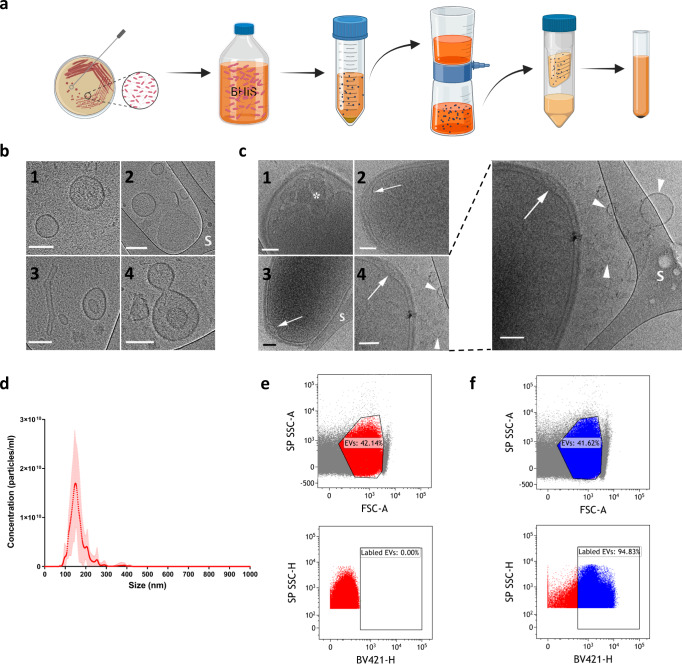
*B. longum* AO44 extracellular vesicles isolation and characterization. **a** Illustration of the extracellular vesicles isolation process. **b** Cryo-TEM images of different bacteria-released EVs from the same sample. ‘S’ in ‘b’ denotes the TEM perforated support film. All bars correspond to 100 nm. **c** Cryo-TEM images of the bacteria in various stages of EV formation: (1) The tip of a bacterium. Asterisk indicates possible forming EVs seen in projection through the cell wall. (2) An EV forming between the cell wall and capsule (arrow). (3) A fully formed EV between the cell wall and the capsule (arrow); ‘S’ denotes the TEM perforated support film. (4) The tip of the bacterium after release of an EV; an arrow points to the distorted cell wall; arrowheads point to released EVs. An enlargement of (4) is added on the right. Scale bars correspond to 100 nm. **d** Size distribution and concentration (1e10) of *B. longum* extracellular vesicles as measured in NanoSight. Error bars represent s.d. **e**, **f** *B. longum* extracellular vesicles fluorescently labeled by D-amino acid labeling, detected in flow cytometry. **e** unlabeled vesicles and **f** labeled vesicles. Figure 2a was created with BioRender.com.

According to the chemical characteristics of CHCl_3_, this fraction contains mostly hydrophobic molecules, such as bacterial cell membranes. Since both the CHCl_3_ fraction and the concentrated supernatant fraction had a similar effect on IL-10 secretion, and EVs are composed of bacterial cell membranes secreted to the conditioned media (i.e., present in the supernatants), we chose to focus on characterizing EVs as the active bacterial entity. EVs of *B. longum* AO44 were isolated through a series of filtrations and ultracentrifugation (Fig. 2a) to achieve EVs in pellets. As a control, BHIS media with no bacteria was passed through the same process of filtrations and ultracentrifugation to confirm that the immune effect is derived from a bacterial entity and not from the concentrated media itself. The EVs were characterized (Fig. 2b–d) by morphology and size. A representative Cryo-TEM image confirms the presence of the vesicles in the ultracentrifuged pellet and demonstrates their morphology (Fig. 2b). Moreover, EVs were identified budding from the bacterial cell membrane as well as surrounding the bacterial cells in their growth media, using Cryo-TEM (Fig. 2c). The size distribution of the EVs was measured using NanoSight, with most vesicles being the size of 150 nm (Fig. 2d). To verify that the EVs were synthesized by *B. longum*, the bacteria were grown in the presence of fluorescent D-amino acids to label newly synthesized proteins, and specifically the peptidoglycans of the bacteria. Metabolically active bacteria incorporate the fluorescent D-amino acids in order to synthesize proteins. Metabolic labeling by fluorescent D-amino acids is a method that allows labeling of newly synthesized bacterial EVs during their production, without the need to label them after extraction, a step that might leave traces of the fluorescence markers that can cause non-specific labeling artifacts. The labeled vesicles were identified and differentiated from the unlabeled vesicles by a specific flow cytometry designed to detect small particles (Fig. 2e, f).

### *B. longum* EVs proteomics characterization

**Fig. 3:**
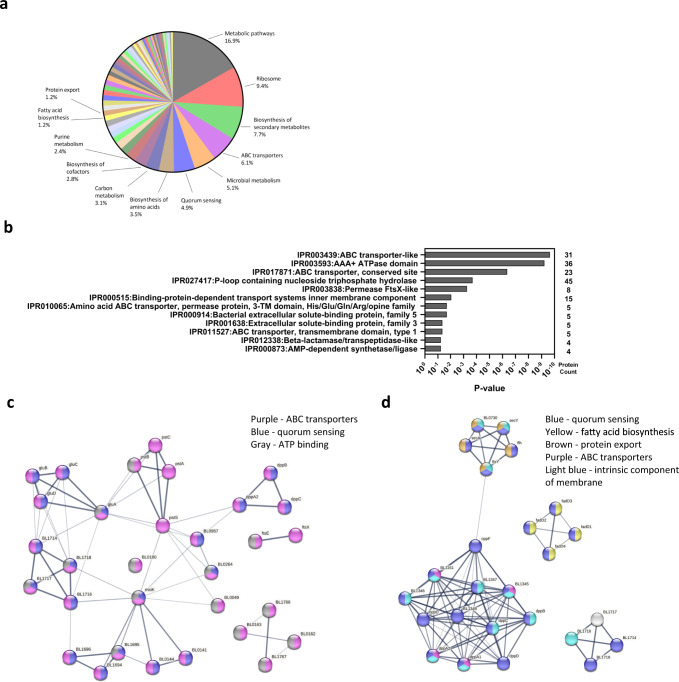
Annotation enrichment of the protein profile of *B. longum* extracellular vesicles. **a** KEGG pathways enriched in *B. longum* EVs. **b** Enriched domain and protein families’ categories of the INTERPRO database (*P*-value and protein count) as analyzed by the DAVID Bioinformatics Resources (LHRI/ADRD at Frederick National Laboratory). The enrichment was done against the bacterial genome background. **c** STRING analysis and interactions map of the ABC transporters cluster as annotated by KEGG pathway database (purple—ABC transporters, blue—quorum sensing, gray—ATP binding). **d** STRING analysis and interactions map of the quorum sensing proteins as annotated by KEGG pathway database (blue—quorum sensing, yellow—fatty acid biosynthesis, brown—protein export, purple—ABC transporters, light blue—intrinsic component of membrane).

*B. longum* AO44 EVs were analyzed for their proteome content through liquid chromatography-tandem mass spectrometry. Four hundred and sixty-three proteins were identified and quantified in all three repeats. The enriched KEGG pathways are presented in percentages in Fig. 3a and Supplementary Table 1c. The three largest groups of proteins belonged to: metabolic pathways (16.9%), Ribosomes (9.4%), and biosynthesis of secondary metabolites (7.7%), the fourth largest group was ABC transporters (6.1%), and the sixth largest group was quorum sensing (4.9%). Moreover, ABC transporters were the most significantly enriched category according to the INTERPRO database with protein counts of 31 and a *p*-value of 1e−10 (Fig. 3b and Supplementary Table 1b). INTERPRO confirmed that other protein categories related to ABC transporters are enriched in *B. longum* AO44 EVs, including transmembrane permease proteins, nucleotide-binding proteins, and highly specific periplasmic solute-binding proteins (Fig. 3b). STRING analysis and interactions map of these ABC transporters subset and the quorum sensing subset as analyzed by KEGG are presented in Fig. 3c, d. To note, the ABC transporters subset also contained proteins belonging to the quorum sensing pathway and an ATP binding pathway (Fig. 3c). The quorum sensing subset included proteins involved in fatty acid biosynthesis, protein export, ABC transporters and proteins that are intrinsic components of the membrane (Fig. 3d). All proteins identified in the vesicles are listed in Supplementary Table 1 (Supplementary Table 1a includes the whole list of integral membrane components, Supplementary 1b includes the INTERPRO analysis, and Supplementary 1c the KEGG analysis).

**Fig. 4:**
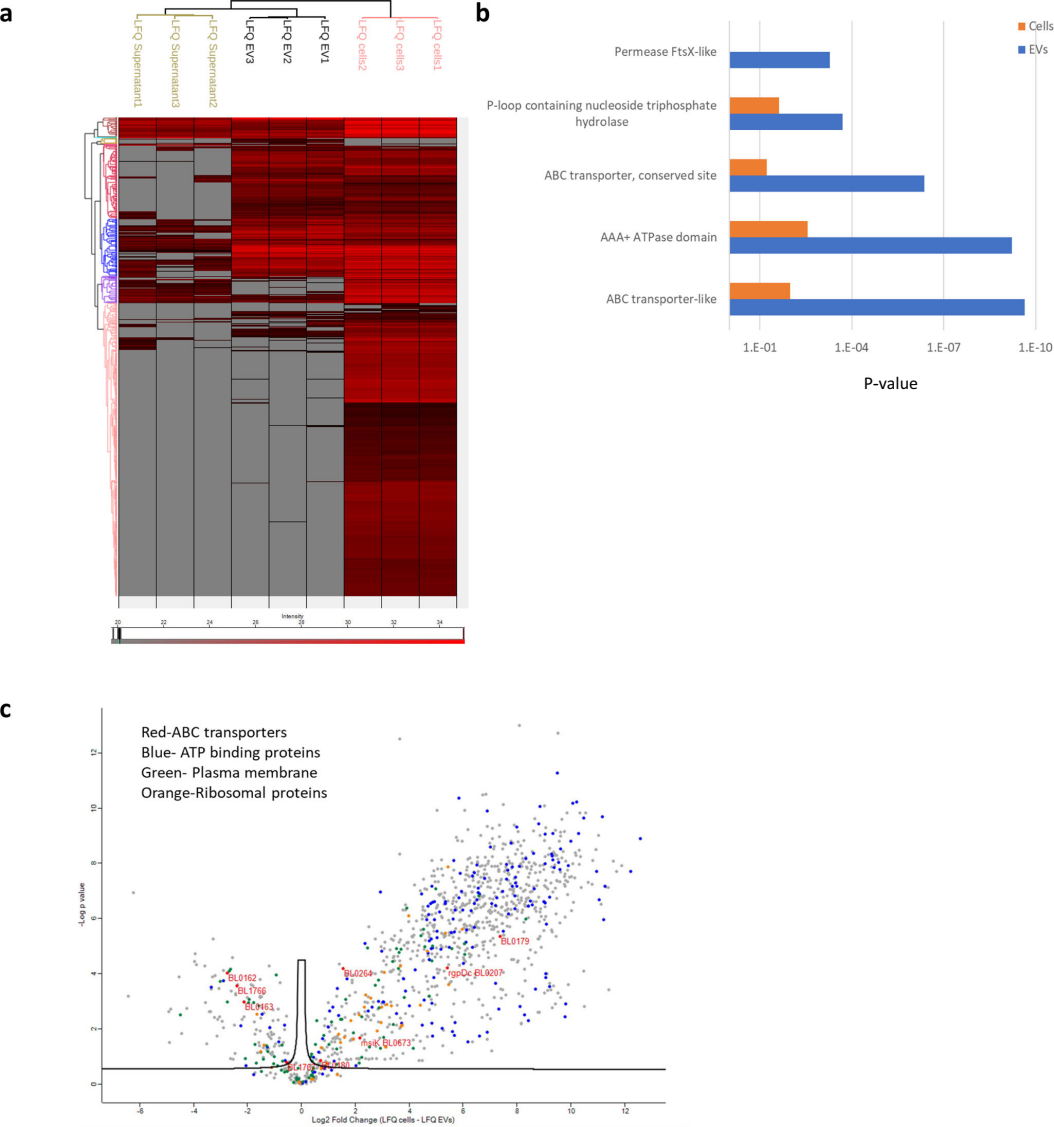
Protein profile of *B. longum* cells, extracellular vesicles, and supernatants. **a** Heat map representation of the protein intensities of bacterial cells, EVs, and supernatants samples, as analyzed by the Perseus software. This unsupervised hierarchical clustering was performed using Euclidean distance analysis. **b** Enriched domain and protein families’ categories of the INTERPRO database (*P*-values) as analyzed by the DAVID Bioinformatics Resources (LHRI/ADRD at Frederick National Laboratory) comparing bacterial cells and EVs. The enrichment was done against the bacterial genome background. **c** Volcano plot representation of the Differential proteins identified in the bacterial cells (right) and the EVs (left). Red—ABC transporters, blue—ATP binding proteins, green—Plasma membrane, orange—Ribosomal proteins.

In order to validate that the protein profile of the EVs differs from the protein profile of the bacterial cells and supernatants, proteomics analysis was performed also on the bacterial cells and supernatants (Supplementary Table 2). The bacterial cells’ protein intensities profiles were compared to the EVs data and to the proteins analyzed from the supernatants after ultracentrifugation. Replicates from each group were clustered together by unsupervised clustering, represented in a heat map (Fig. 4a). Multiple intense proteins were identified in the bacterial cells’ proteomics but not in the EVs, and in the EVs but not in the supernatants, indicating that the EVs contain a specific protein content, which is unique to the EVs and different from the total protein content of the cells as well as from the secreted protein content. ABC transporters proteins and domains were significantly enriched in EVs compared to bacterial cells according to the INTERPRO database (Fig. 4b). Differential proteins identified in the bacterial cells (right) and the EVs (left) are represented in the volcano plot (Fig. 4c).

### *B. longum* AO44 EVs have an anti-inflammatory effect on the immune system

**Fig. 5:**
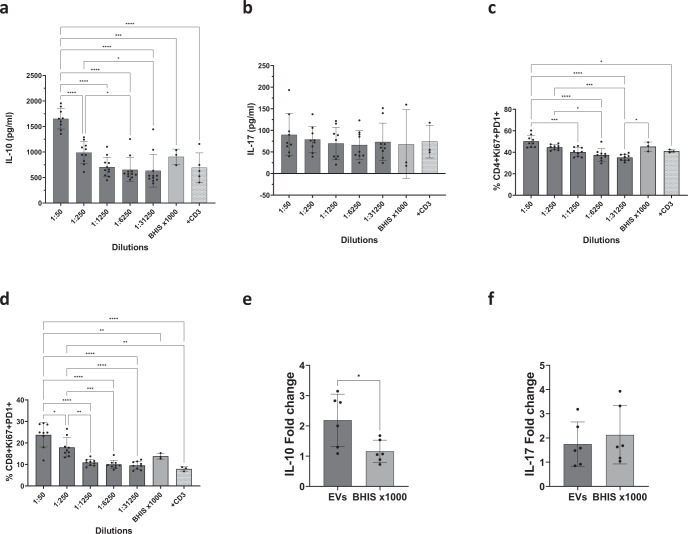
Immune-modulatory activity of *B. longum* AO44 extracellular vesicles. **a** IL-10 concentrations and **b** IL-17 concentrations in splenocytes media after exposure to anti-CD3; or anti-CD3 combined with concentrated BHIS (light gray, 1:50 dilution) or descending dilutions of extracellular vesicles. **c** CD4+ Ki67+ PD1+ cell frequency out of total CD4 cells after exposure to anti-CD3; anti-CD3 combined with concentrated BHIS (1:50 dilution) or descending dilutions of extracellular vesicles. **d** CD8+ Ki67+ PD1+ cell frequency out of total CD8 cells after exposure to anti-CD3 or anti-CD3 combined with concentrated BHIS (1:50 dilution) or descending dilutions of extracellular vesicles. **e**, **f** Fold change (compared to control) of IL-10 (**e**) or IL-17 (**f**) concentrations in DC-CD4+ T cells media exposed to either *B. longum* EVs or concentrated BHIS. Each dot represents a biological repeat out of three independent experiments. **a**–**d** Ordinary one-way ANOVA, **e**, **f** unpaired t test. *P < 0.05, **P < 0.01, ***P < 0.001, and ****P < 0.0001. Error bars represent s.d.

EVs were introduced to SPF mouse splenocytes in descending concentrations and both IL-10 and IL-17 concentrations in the cells’ media were measured. IL-10 concentration was highest at the lowest dilution of EVs (1:50) compared to all other dilutions and controls. Concentrations of IL-10 decreased in a nonlinear fashion when vesicles were diluted to a non-effective concentration at 1:1250 dilution (Fig. 5a). On the contrary, IL-17 concentrations were not affected by the vesicles in all concentrations (Fig. 5b). CD8+ Ki67+ PD1+ and CD4+ Ki67+ PD1+ cell frequencies were measured by flow cytometry to assess cell proliferation and activation. Both CD8+ Ki67+ PD1+ and CD4+ Ki67+ PD1+ cell frequencies were higher when the cells were exposed to the lowest dilution of vesicles (1:50), with CD8+ Ki67+ PD1+ being slightly more significant compared to other dilutions and the controls (Fig. 5c, d). To further investigate the anti-inflammatory response of antigen-presenting cells and T cells to *B. longum* EVs, DC-CD4+ T cells were co-cultured in the presence of the bacterial EVs. IL-10 levels were significantly higher in cells activated with EVs of *B. longum* compared to cells activated with concentrated BHIS (x1000, Fig. 5e). IL-17 fold change showed no significant differences, and even a slight decrease, in cells activated with EVs compared to cells activated with concentrated BHIS (x1000, Fig. 5f). Both IL-10 and IL-17 induction in the DC-CD4+ T cells co-culture aligned with the induction in the whole splenocytes assay.

## Discussion

The gut microbiota has major potential effects on our health, so much that it can be considered a gold mine for therapeutic molecules derived from the bacteria^19–21^. A major route of bacterial-induced therapeutic effects on the host is via bacterial communication with the host immune system^6–9,16,49^. For example, SCFAs produced by gut bacteria can induce anti-inflammatory effects on the host via induction of T-regs^50^. However, the modes of such commensal-host communications and the bacterial immune-modulatory molecules involved, are still largely unknown. One possible mode of gut microbe-host interactions is via the secretion of bacterial vesicles, which can reach host cells, potentially even in distant sites. Indeed, an outer surface polysaccharide (PS) of the Gram-negative bacteria *Bacteroides fragilis* is communicated to host cells via bacterial outer membrane vesicles (OMVs), alleviating experimental colitis in mice^51^. Studies on extracellular vesicles (EVs) of Gram-positive gut bacteria are emerging, however, are still relatively scarce, compared to studies on Gram-negative OMVs^46,47^. Specifically, a few recent studies demonstrated the physiological effects of EVs produced by bacteria from the *Bifidobacterium* genus. Of this genus, *Bifidobacterium longum* is a common Gram-positive gut commensal with anti-inflammatory and immune-modulatory effects.

Here we chose to study a newly isolated human gut-derived *B. longum* strain AO44, aiming to characterize its immune-modulatory entities. We first performed chemical fractionation of bacterial cells and supernatants of *B. longum* AO44 to discover the chemical characteristics of the active immune-modulatory bacterial compounds. Two rich growth media were chosen (BHIS and GAM), which are prevalently used to grow *Bifidobacterium* species. We performed CHCl_3_:MeOH:H_2_O fractionations, which separates molecules into crude hydrophobic and hydrophilic fractions, organic and polar fractions. Since bacteria from the *Bifidobacterium* genus were shown to induce mostly anti-inflammatory effects^38^, we screened for induction of the anti-inflammatory cytokine IL-10 by the active fractions. Both the concentrated supernatant fraction and the CHCl_3_ cell fraction had the highest effect on IL-10 secretion by immune cells, both in GAM and BHIS media. The CHCl_3_ nonpolar organic fraction contains hydrophobic molecules such as membrane parts and lipids. Therefore, we speculated that the active component is a cellular substance found within both. Since the supernatant was separated from the bacterial cells by centrifugation, hydrophobic membrane parts found are likely to be cell debris or EVs secreted by the bacterial cells to the media. Therefore, we next isolated EVs from bacteria grown in BHIS and confirmed their ability to induce the secretion of IL-10 but not IL-17 in whole splenocytes culture, which emphasizes their anti-inflammatory effect.

We further explored their additional potential immune effects and identified that these same EVs are also capable of increasing CD4+ Ki67+ PD1+ and CD8+ Ki67+ PD1+ cell frequencies compared to the control. Upregulation of CD4+ Ki67+ PD1+ and CD8+ Ki67+ PD1+ indicate activation (PD1+) and proliferation (Ki67+) of both CD4 and CD8 cells, which aligns with the induction of IL-10. For a more mechanistic insight into the EVs anti-inflammatory effects on host immune cells we explored their immune-modulatory effects in DC-CD4+ T cells co-culture. Here, as with the whole splenocytes, the EVs induced IL-10 secretion and not IL-17. This again points to their anti-inflammatory effects and identifies DCs and CD4+ T cells as major players in this interaction. Once we identified the EVs immune-modulatory effects, we characterized their contents by proteomics. The proteomics analysis revealed unique proteins content in the EVs which differs from the protein content of whole bacterial cells and secreted proteins in the supernatant. Specifically, enrichment of ABC transporters and quorum-sensing proteins was observed in the EVs. Bacterial high-affinity transport systems are involved in the active transport of solutes across the cytoplasmic membrane, including transmembrane permease proteins, nucleotide-binding proteins, and highly specific periplasmic solute-binding proteins, all present in the vesicles. To note, ABC transporters and extracellular solute-binding proteins (including “family 5”) were enriched in the EVs of another *B. longum* strain which was shown to alleviate allergies^46^. Moreover, quorum sensing proteins were previously shown to be enriched in Gram-negative OMVs, promoting bacterial biofilm formation^52,53^. Lastly, we demonstrate specific fluorescence labeling of the bacterial vesicles, which can enable future studies on EVs-host interactions. To summarize, we characterized by morphology, size, content, and immune-modulatory activities EVs of a newly isolated gut-derived *B. longum*. Our results demonstrating EVs mediated immune cells’ activation can lead to future studies assessing their effects in vivo, in health and disease, and considering these specific EVs as potential future therapeutics.

## Methods

### Strain culture

*B. longum* AO44 was thawed on Brain Heart Infusion agar plates (BHI, BD BBL^TM^) supplemented with 5 µg/ml hemin (Alfa Aesar) in 1 N NaOH and 2.5 µg/ml vitamin K (Thermo Fisher Scientific) in 100% EtOH (previously referred to as BHIS), at 37 °C in an anaerobic chamber, 85% N_2_, 10% CO_2_, 5% H_2_ (COY).

### Fractionation

*B. longum* AO44 was pre-cultured at 37 °C under anaerobic conditions. First, in 10 ml of BHIS or GAM in ~20 ml Hungate tube, and then inoculated by 0.5% v/v to 900 mL of BHIS or GAM in 1 L glass bottle at 37 °C under anaerobic conditions. After 4 days incubation at 37 °C, the supernatant and cells were separated by centrifugation (7500 × *g*, Thermo Fisher Scientific Sorvall R6 centrifuge with 1Lx4 bottle rotor).

Supernatant fractionation—10x supernatant fraction (i.e., crude concentrated supernatant) was prepared by evaporating the centrifuged supernatant to 1/10 volume by rotary evaporator. Supernatant-methanol fractions were prepared as follows: 10 ml of the centrifuged supernatant was first lyophilized to dryness. 30 ml of methanol was added to the dry residue. After mixing by spatula and by sonication (15 min), the mixed solution was left overnight at room temperature (RT) to let the molecules be fully extracted in methanol. The methanol solution was carefully removed from the insoluble residues. This methanol fraction (30 ml) was concentrated in vacuo to dryness and re-dissolved in 1 ml of dimethyl sulfoxide (DMSO). The supernatant-water fraction was prepared as follows: the insoluble residues from the above process were dissolved in 1 ml of water.

Cell pellet fractionation—cell-chloroform fraction was prepared as follows: cell pellets from the 1 L culture were soaked in 300 ml of chloroform:methanol = 1:1 (v/v). After mixing by spatula and by sonication (15 min), the mixed solution was left overnight at RT to let the molecules be fully extracted in organic solvent. The extract was removed from cell pellets, filtrated, and evaporated in vacuo to dryness. The dry residue was then added 30 ml of chloroform, mixed by spatula and by sonication (15 min), and left overnight at RT to let the molecules fully extracted into organic solvent. The chloroform solution was removed from the insoluble residues, concentrated in vacuo, and subsequentially re-dissolved in 3 ml of DMSO. Cell-methanol fractions were prepared as follows: the insoluble residues from the above cell-chloroform extraction process were dissolved in 30 ml of methanol, mixed by spatula and by sonication (15 min), and left overnight at RT for complete molecular extraction into the organic solvent. The methanol solution was removed from the insoluble residues, concentrated in vacuo, and re-dissolved in 3 ml of DMSO.

### Vesicles isolation

*B. longum* AO44 was grown in 10 ml BHIS at 37 °C under anaerobic conditions overnight. Bacterial culture was then diluted 1:40 to 200 ml of BHIS and grown for 8 h at 37 °C under anaerobic conditions, a second dilution of 1:20 was done and bacteria were grown for 16 h at 37 °C under anaerobic conditions. The culture was centrifuged at 8000 × *g* for 30 min, then, filtered twice through a 0.45-µm-pore size filter and a 0.22-µm-pore size filter. The filtered supernatant was then concentrated using Centricon® Plus-70 Centrifugal Filter Units (Merck) with a 100-kDa-exclusion. The remaining supernatant was ultracentrifuged at 100,000 × *g* for 1 h at 4 °C to obtain the membrane vesicle pellet. After ultracentrifugation, the upper liquid was discarded and the vesicle pellet was re-suspended in 1 ml sterile Dulbecco’s Phosphate Buffered Saline (PBS), filtered with a 0.22-µm-pore size filter and frozen at −80 °C. Vesicle size distribution was measured using NanoSight.

### Splenocytes culture

Spleens were harvested from C57BL/6 SPF mice and were mechanically disrupted on a 40 µm cell strainer with 1 ml of ACK lysing buffer (Thermo Fisher Scientific) for 1–2 min. Cells were then transferred to 20 ml ice-cold RPMI (Sartorius) medium and centrifuged for 10 min at 4 °C and 300 × *g*. Cells were washed twice with 10 ml of ice-cold RPMI, counted and diluted with 37 °C RPMI to a final concentration of 2 million cells/ml. Cells were then supplemented with 0.05 mM β-Mercaptoethanol (Merck) and anti-CD3 (0.5 µg/ml, 145-2C11, BioLegend) for suboptimal activation of the T cells. 100 µl of cells were plated in each well of a 96-well plate and were added with 100 µl of diluted fractions (1:250 dilution) or vesicles (1:50–1:31,250 dilution). Cells and their media were collected 5 days later for flow cytometry and ELISA analysis.

### DC-CD4+ T cells co-culture

Spleens were harvested from C57BL/6 SPF mice. For the extraction of DC, spleens were injected with collagenase ii in a concentration of 1 mg/ml in RPMI medium, incubated at 37 °C for 30 min, and then mechanically disrupted on a 40 µm cell strainer. For the extraction of CD4+ T cells, spleens were mechanically disrupted on a 40 µm cell strainer with 1 ml PBS containing 2% fetal bovine serum (FBS). Cells were then washed twice with 10 ml PBS or Hanks’ Balanced Salt Solution (HBSS) containing 2% FBS and 1 mM EDTA, and centrifuged for 5 min at room temperature and 300 × *g*. Cells from each spleen were either used to isolate DC using EasySep™ Mouse Pan-DC Enrichment Kit (STEMCELL Technologies Inc.) or CD4+ T cells using EasySep Mouse CD4+ T Cell Isolation Kit (STEMCELL Technologies Inc.) according to the manufacturer’s instructions. 50 µl of isolated DC were plated in a 96-weel plate in RPMI medium at a final concentration of 400,000 cells/ml. 50 µl of isolated CD4+ T cells were plated in the same 96-weel plate in RPMI medium at a final concentration of 2 million cells/ml. Cells were then supplemented with 0.05 mM β-Mercaptoethanol (Merck) and anti-CD3 (0.5 µg/ml, 145-2C11, BioLegend) for suboptimal activation of the CD4+ T cells. DC-CD4+ T cells co-culture was then added with 100 µl of diluted vesicles (1:50 dilution) and concentrated BHIS as a control (1:50 dilution). Cells media were collected 3 days later for ELISA analysis.

### Cryo-transmission electron microscopy (cryo-TEM)

Cryo-TEM specimens were prepared in a controlled environment vitrification system (CEVS)^54^, at a controlled temperature (25 °C) and 100% relative humidity. A 4 μL drop of solution was applied to a carbon-coated perforated film supported on a TEM grid (Lacey Formvar/carbon films on 200 mesh copper grid, Ted Pella Inc., Redding, USA), held by tweezers, inside the CEVS. The grids were earlier plasma-etched in a PELCO EasiGlow glow-discharger (Ted Pella Inc., Redding, USA) to increase their hydrophilicity. Excess liquid was blotted twice from the back of the grid using filter paper, forming a thin film suitable for imaging. The solution was then vitrified by quickly plunging the grid into liquid ethane at its freezing point (−183 °C). The specimens were stored in a liquid nitrogen dewar until imaging. The specimens were imaged using a Thermo-Fisher Scientific Talos 200C, a high-resolution TEM operated at 200 kV and equipped with a field emission gun (FEG), and a Falcon III direct-imaging camera. The transfer of specimens into the microscope was done using a Gatan 626 cryo-holder kept at −180 °C. All images were recorded by low-dose imaging, to minimize electron-beam radiation damage, and with a Volta phase-plate (VPP), to enhance image contrast.

### D-amino acid labeling and flow cytometry of small particles

Bacteria were grown overnight at 37 °C under anaerobic conditions in an anaerobic chamber in BHIS media supplemented with HADA (0.8 mM, Tocris, Bio-Techne), total volume of 10 ml^55^. Labeled bacteria were separated from the media by centrifugation for 5 min at 7500 × *g*. Vesicles were then isolated as described above and detected by BD FACSymphony^TM^ A1, a flow cytometry analyzer with a small particle detector which is able to detect particles as small as 90 nm. Voltages used: FS - 550, SS - 650, SP SSC - 450, BV421 - 460.

### Flow cytometry

A constant panel of antibodies was used for consistency. The panel included antibodies against CD4 (RMA-5), CD8 (53-6.7), TCRß (H57-597), CD19 (6D5), Ki67 (16A8), PD-1 (29F.1A12, all from BioLegend). For intracellular staining of transcription factors, cells were stained for surface markers and fixed in Fix/Perm buffer (eBioscience) for 30–60 min at RT, and permeabilized in permeabilization buffer (eBioscience) at RT for 30 min in the presence of antibodies. Cells were acquired with a BD BioSciences^®^ LSRFortessa, and analysis was performed with Kaluza^®^ Analysis Software. The concentration, clone, and source of antibodies were kept constant to ensure consistency in staining.

### ELISA assay

IL-10 and IL-17 concentrations in splenocytes and DC-CD4+ T cells media were measured using Mouse IL-10 and IL-17 ELISA MAX^TM^ Standard Kit (BioLegend) following the manufacturer’s instructions. ELISA limits of detection: IL-10—31.5–2000 pg/ml, IL-17—15.6–1000 pg/ml.

### Proteolysis and mass spectrometry analysis

The EVs and supernatants samples were dissolved in 10 mM Dithiothreitol (DTT), 100 mM Tris, and 5% sodium dodecyl sulfate (SDS), sonicated and boiled at 95 °C for 5 min and precipitated in 80% acetone. The protein pellets were dissolved in 9 M Urea and 400 mM ammonium bicarbonate, then reduced with 3 mM DTT (at 60 °C for 30 min), modified with 10 mM iodoacetamide in 100 mM ammonium bicarbonate (at RT 30 min in the dark) and digested in 2 M Urea, 25 mM ammonium bicarbonate with modified trypsin (Promega), overnight at 37 °C in a 1:50 (M/M) enzyme-to-substrate ratio. The tryptic peptides were desalted using a homemade C18 stage tip, dried and re-suspended in 0.1% formic acid. The peptides were resolved by reverse-phase chromatography on 0.075 × 300-mm fused silica capillaries (J&W) packed with Reprosil reversed phase material (Dr Maisch GmbH, Germany). The peptides were eluted with a linear 60 min gradient of 5% to 28%, 15 min gradient of 28% to 95%, and 15 min at 95% acetonitrile with 0.1% formic acid in water at flow rates of 0.15 μl/min. Mass spectrometry was performed by Q Exactive HF mass spectrometer (Thermo Fisher Scientific) in a positive mode (*m*/*z* 300–1800, resolution 60,000 for MS1 and 15,000 for MS2) using repetitively full MS scan followed by high collision induced dissociation (HCD, at 27 normalized collision energy) of the 18 most dominant ions (>1 charge) selected from the first MS scan. A dynamic exclusion list was enabled with an exclusion duration of 20 s. The mass spectrometry data was analyzed using the MaxQuant software 1.5.2.8^56^ for peak picking and identification using the Andromeda search engine, searching against *Bifidobacterium longum* proteome from the Uniprot database with a mass tolerance of 6 ppm for the precursor masses and 20 ppm for the fragment ions. Oxidation on methionine and protein N-terminus acetylation were accepted as variable modifications and carbamidomethyl on cysteine was accepted as a static modification. Minimal peptide length was set to six amino acids and a maximum of two miscleavages was allowed. The data was quantified by label-free analysis using the same software. Peptide- and protein-level false discovery rates (FDRs) were filtered to 1% using the target-decoy strategy. Protein tables were filtered to eliminate the identifications from the reverse database, common contaminants, and single peptide identifications^57^.

For the bacterial cells samples, the peptides were eluted with linear 180 min gradient of 5% to 28%, 15 min gradient of 28% to 95%, and 25 min at 95% acetonitrile with 0.1% formic acid in water at flow rates of 0.15 μl/min. Mass spectrometry was performed by Exploris 480 mass spectrometer (Thermo) in a positive mode (*m*/*z* 350–1200, resolution 120,000 for MS1 and 15,000 for MS2) using repetitively full MS scan followed by high collision induces dissociation (HCD, at 27 normalized collision energy) of the 30 most dominant ions (>1 charges) selected from the first MS scan. A dynamic exclusion list was enabled with exclusion duration of 30 s. MS data analysis was done similar to the EVs samples.

### DNA extraction and whole-genome sequencing

*B. longum* strain AO44, human isolate, was used in this study^16^ (Brigham and Women’s Hospital). The genomic DNA was extracted using ZymoBIOMICS DNA Miniprep Kit (Zymo Research). DNA was sequenced by Illumina MiSeq PE 2x150 and the assembly method used was SPades v3.15.3.

### Statistical analysis

Mass Spectrometry analysis of the identification and quantization of the vesicles’ proteome content was done using Perseus 1.6.10.43 software. All other statistical analyses were done using Prism-GraphPad.

### Reporting summary

Further information on research design is available in the Nature Research Reporting Summary linked to this article.

## Supplementary information

Supplemental table 1 and 2

Reporting Summary

## Data Availability

The data analyzed in this study are available within the article and its supplementary tables file. The *B. longum* whole-genome sequence has been deposited to GenBank, accession number PRJNA908295. The mass spectrometry proteomics data have been deposited to the ProteomeXchange Consortium via the PRIDE partner repository, dataset identifier PXD038667. Additional data are available from the corresponding author upon request.
